# The facilitators and barriers to exercise in the Noongar Aboriginal population in Perth, Australia

**DOI:** 10.1093/heapro/daac023

**Published:** 2022-03-28

**Authors:** Tuguy Esgin, Deborah Hersh, Kevin Rowley, Rona Macniven, Alan Crouch, Mark Halaki, Robert Newton

**Affiliations:** 1 Discipline of Exercise and Sport Science, School of Health Sciences, The University of Sydney, Sydney, New South Wales 2006, Australia; 2 School of Medical and Health Sciences, Edith Cowan University, 270 Joondalup Drive, Joondalup, Western Australia 6027, Australia; 3 Exercise Medicine Research Institute, Edith Cowan University, 270 Joondalup Drive, Joondalup, Western Australia 6027, Australia; 4 School of Management and Governance UNSW Business School, University of New South Wales Sydney, Kensington, New South Wales 2052, Australia; 5 Curtin School of Allied Health, Curtin University, Kent Street, Bentley, Perth, Western Australia 6102, Australia; 6 Onemda Aboriginal and Torres Strait Islander Health, Wellbeing, Equity and Healing Unit VicHealth Koori Health Unit, University of Melbourne, 207 Bouverie St, Carlton Victoria 3053, Australia; 7 School of Population Health, Faculty of Medicine and Health, University of New South Wales Sydney, Kensington, New South Wales 2052, Australia; 8 Faculty of Medicine, Health and Human Sciences, Macquarie University, Sydney, New South Wales 2019, Australia; 9 Department of Rural Health, The University of Melbourne, Ballarat Campus, 806 Mair St, Ballarat, Victoria 3350, Australia

**Keywords:** Noongar Community, exercise, adherence, physical activity, Aboriginal, Australia

## Abstract

Indigenous Standpoint Theory forms the epistemological foundation for this study and methodological choices were made within this theoretical framework to ensure culturally responsive research processes that engaged the Indigenous agenda of self-determination and rights. The objectives of this research were to determine: (i) Indigenous perceptions of the facilitators and barriers to exercise; (ii) The potential feasibility and sustainability of an exercise intervention. In this context, Participatory Action Research methods were used to design the data-gathering instrument for the study—a questionnaire, co-designed with the Noongar Aboriginal community of Perth, Western Australia. This self-administered questionnaire, distributed to participants by email, post and manual delivery, sought to elicit the factors that impact uptake and retention of regular exercise activities. Questionnaire data included individual demographic detail and specific question responses on labelled 5 point Likert Scales. Specific question responses were tabulated by Likert Scale label category and the response distribution for each question was enumerated. Simple descriptive statistics (measures of central tendency and variance) were used to characterize the data set and the Chi squared test was used to evaluate frequency differences between males and females. A total of 133 participants (71 females) completed the questionnaire. The results indicated that people valued exercise. The most common barriers indicated by participants were exercising with an injury (63%), changing diet (58%), finding time to exercise every day (55%) and exercising the next day with pain from exercising the day before (54%). A larger proportion of males (34%) than females (24%) reported greater ease in finding time to exercise every day (*p* < 0.05). Facilitators mainly related to the potential social and community benefits of exercising with other people, preferably in small groups, and the importance of a culturally secure venue. These findings shed light on what a culturally secure exercise programme might involve for the Noongar community. As this may have implications for other Aboriginal and Torres Strait Islander and international First Nations’ Peoples, more focused research is needed on the place of traditional physical activities and the nature of culturally secure exercise programmes and spaces to enable wider application.

## INTRODUCTION

Reducing the incidence and prevalence of preventable chronic and cardiometabolic diseases involves taking action on a range of determinants and risk factors. Studies undertaken with and by First Nations researchers in several global contexts have highlighted the impact on health outcomes of social determinants. These include under-investment in Indigenous health infrastructure and amenities, lack of access to relevant health services, lack of cultural sensitivity and continuity of care, loss of land, language and culture, racism and discrimination (Harris *et al.*, 2006; Kritharides *et al.*, 2010).

For Indigenous People in Australia, significant health gains are likely to be achieved by addressing those determinants that impact access to culturally secure health services and activities and by tackling those risk factors that mediate the bulk of preventable chronic disease (Gray *et al.*, 2013; Merone *et al.*, 2020). Appropriate forms and levels of exercise are an evidence-based component of strategies to reduce the burden of preventable chronic and cardiometabolic disease. It is, therefore, important to identify effective and sustainable ways to increase exercise levels among Aboriginal communities. Such a goal also ties in closely with the message that ‘exercise is medicine’ (Sallis, 2009).

Exercise is a subset of physical activity that is planned, structured and repetitive to improve physical fitness (Caspersen *et al.*, 1985). Increasing the level of exercise is key to improving Indigenous health outcomes. Increasing cardiorespiratory fitness and muscular strength has been demonstrated to reduce avoidable chronic diseases (Esgin, 2019). However, cultural values and traditions influence participation in exercise by Indigenous communities (Dahlberg *et al.*, 2018). Understanding community environmental influences on exercise is essential to designing interventions to increase exercise participation (Kirby *et al.*, 2007). Many factors can contribute to community environmental influences that pertain to the built environment, including footpaths, parks, pools and gyms. However, a promising theme of cultural relevance is that engaging in exercise has the potential to enhance family ties in the community (Marshall *et al.*, 2008).

This suggests that exercise may have a strong social function in Indigenous communities in addition to providing physical health benefits, consistent with a recent review that physical activity and sport can confer broader social benefits (Macniven *et al.*, 2019). The aim of the current study was to examine the facilitators and barriers to engaging in exercise for the Noongar Aboriginal community in the Perth metropolitan area.

Cultural safety is defined as ‘an environment which is safe for people; where there is no assault, challenge or denial of their identity, of who they are and what they need’ (Williams, 1999). It is about shared respect, shared meaning, shared knowledge and experience, of learning together with dignity, and ‘truly listening’ (p. 213). A more detailed description of the process of development of the questionnaire to be culturally safe has been described elsewhere (Esgin *et al.*, 2019).

## METHODS

Indigenous Standpoint Theory (IST), in which the researcher establishes a ‘positionality’ within their ancestors’ culture and knowledge, forms the epistemological foundation for this study. Methodological choices were made within this theoretical framework to ensure culturally responsive research processes that engaged the Indigenous agenda of self-determination and rights (Foley, 2006; Smith, 2021). In this context, Participatory Action Research methods were used to embed culturally responsive community co-design and co-production processes across the study, specifically in the development and distribution of a self-administered questionnaire tool (Esgin *et al.*, 2019).

Participation in the study was voluntary and no incentives were offered to the respondents. The study procedure was fully explained to each participant and written informed consent was obtained from all participants before prior to administration of the study questionnaire.

The 30-item questionnaire was designed, piloted and administered to determine the facilitators and barriers to exercise in the Noongar community in 2014 (Esgin *et al.*, 2019). This instrument was designed in consultation with key community stakeholders, Noongar Elders and Noongar Academics, facilitated by the first author (TE), a member of the Noongar community. The questionnaire was self-administered (see Supplementary information) and included a series of questions about participant demographics and health, barriers to exercise, exercise preferences and environment, and exercise advice and types. The questionnaire was distributed to the Noongar community by email, post and manual delivery to individuals in relevant organizations.

### Participants

Eligible participants were those aged 18–60 years who identified as Australian Aboriginal, living in the Perth metropolitan area. With approximately 85% of the participants identifying as Noongar. Participants were recruited through established networks at Aboriginal educational institutions, primary health centres, government departments and the private sector organizations, selected on the criterion that they employed a large number of Indigenous people or provided services to large numbers of individuals in the Noongar community. These participating organizations included Derbarl Yerrigan Health Service, Marr Mooditj Foundation and the Centre for Aboriginal Studies at Curtin University.

### Analysis

Specific question results were tabulated by Likert Scale label category and analysed for frequency. All statistical analyses were performed using PASW SPSS version 17.0 statistical software package. Analysis included standard descriptive statistics (measures of central tendency and variance) and prevalence frequencies that were compared by sex using Chi-square tests (or Fisher’s exact test when cell size was *n* < 5). A significance level of *p* < 0.05 was used.

## RESULTS

### Demographic and health

A total of 139 questionnaires were distributed with 137 questionnaires returned, a response rate of 99%. However, four of the returned questionnaires had some missing data and were excluded from the analysis (complete case analysis). The characteristics of the participants are summarized in Table 1.

**Table 1:** Participant demographic and health variables by sex

Variable		Males		Females		*p*
		*N*	%	*N*	%	
Age (*N* = 127)	18–40 years	33	47.8	36	52.2	0.356
	40+ years	23	39.7	35	60.3	
When was the last time you visited your doctor: high blood pressure, diabetes, cholesterol, heart disease? (*N* = 127)	Within 6 months	15	44.1	19	55.9	0.826
	Within 6–12 months	39	41.9	54	58.1	
Has a doctor told you have, or are at risk of: (*N* = 133)	High BP: Yes	12	35.3	22	64.7	0.302
	High BP: No	45	45.5	54	54.5	
	Diabetes: Yes	14	38.9	22	61.1	0.573
	Diabetes: No	43	44.3	54	55.7	
	High cholesterol: Yes	13	40.6	19	59.4	0.770
	High cholesterol: No	44	43.6	57	56.4	
	Heart disease: Yes	4^a^	40.0	6	60.0	0.849
	Heart disease: No	53	43.1	70	56.9	
Body mass index^b^ (*N* = 108)	Healthy weight	10	55.5	8	44.4	0.494
	Overweight	20	52.6	18	47.4	
	Obese	22	42.3	30	57.7	

a Fisher’s exact tests were used where cell sizes were <5. Significance levels did not differ in Fisher’s exact tests.

b No participants were in the underweight BMI category.

Sixty-nine (54%) of participants were age 18–40 years and 71 (54%) were female. Most participants had visited their medical practitioner for high blood pressure, Type 2 Diabetes mellitus (T2DM), hyperlipidaemia (cholesterol) and heart disease in the past 12 months. A minority of participants reported that a medical practitioner has told them they had or were at risk of having high blood pressure (26%); T2DM (27%); high cholesterol (24%) or heart disease (8%). The majority were diagnosed as overweight or obese (83%).

### Barriers to exercise

Participant barriers to exercise are presented in Table 2. The most common barriers indicated by participants were exercising with an injury (63%), changing diet (58%), finding time to exercise every day (55%), and exercising the next day with pain from exercising the day before (54%). A larger proportion of males (34%) than females (24%) reported greater ease in finding time to exercise every day (*p* < 0.05).

**Table 2:** Barriers to exercise by sex

How difficult are the following for you? (*N* = 131)		Male		Female		*p*
		*N*	%	*N*	%	
Changing your diet	Very easy/easy	26	47.3	29	52.7	0.460
	Difficult/very difficult	31	40.8	45	59.2	
Finding time to exercise every day	Very easy/easy	34	58.6	24	41.4	0.002
	Difficult/very difficult	23	31.9	49	68.1	
Motivating yourself to exercise	Very easy/easy	31	50.8	30	49.2	0.115
	Difficult/very difficult	26	37.1	44	62.9	
Exercising with an injury	Very easy/easy	18	50.0	18	50.0	0.586
	Difficult/very difficult	37	44.6	46	55.4	
Putting up with pain associated with exercise	Very easy/easy	29	49.2	30	50.8	0.407
	Difficult/very difficult	28	41.8	39	58.2	
Exercising the next day with pain from exercising the day before	Very easy/easy	26	48.1	28	51.9	0.618
	Difficult/very difficult	31	43.7	40	56.3	
Is exercising vigorously more than 10 min continuously too hard for you?	Never/Occasionally	32	53.3	28	46.7	0.037
	Sometimes/Most of the time/Always	25	35.2	46	64.8	
Is exercise too time-consuming for you?	Never/Occasionally	31	53.4	27	46.6	0.035
	Sometimes/Most of the time/Always	26	35.1	48	64.9	
Is exercising too expensive for you?	Never/Sometimes	39	49.4	40	50.6	0.080
	About half the time/Most times/Always	18	34.0	35	66.0	

More females (46%) than males (25%) reported that exercising vigorously more than 10 min continuously was too hard sometimes/most of the time/always (*p* < 0.05); more females (48%) than males (26%) reported that exercise was sometimes/most of the time/always too time-consuming (*p* < 0.05). Forty per cent of participants indicated that exercising was too expensive about half the time, most times or always, with no significant sex differences.

### Exercise preferences and environment

Participant exercise preferences and environment are presented in Table 3. Participants indicated a range of preferences for exercising on their own, in small or large groups, or no preference. A higher proportion of females (70%) than males (30%) preferred exercising in private (*p* < 0.05). Two-thirds of participants indicated they were happy to exercise indoors or outdoors. Only six participants indicated that they preferred to engage in exercise during a 9–5 working day and in the early morning or early evening; males preferred exercising in public and did not have a time of day preference (all *p* < 0.05). More females (67%) than males (33%) indicated that they felt uncomfortable exercising in groups all, most or some of the time (*p* < 0.05).

**Table 3:** Exercise preferences and environment by sex

Do you prefer to exercise? (*N* = 129)		Males		Females		*p*
		*N*	%	*N*	%	
On your own	All times/mostly	23	51.1	22	48.9	0.116
	Sometimes/never	25	47.2	28	52.8	
	Don’t mind	8	27.6	21	72.4	
In smaller groups (2–3 people)	All times/mostly	18	43.9	23	56.1	0.575
	Sometimes/never	29	46.8	33	53.2	
	Don’t mind	9	34.6	17	65.5	
In larger groups (4–7 people)	All times/mostly	11	47.8	12	52.2	0.823
	Sometimes/never	31	44.3	39	55.7	
	Don’t mind	11	39.3	17	60.7	
In public or private	Public^a^	6	85.7	1	14.3	0.015
	Private	11	29.7	26	70.3	
	Both	35	47.3	39	52.7	
Indoors or outdoors	Indoors	12	42.9	16	57.1	0.819
	Outdoors	7	53.8	6	46.2	
	Both	36	45.6	43	54.4	
Do you enjoy playing games involving sports equipment (i.e. Football, Netball, Rackets, Golf)? (*N* = 130)	All times/mostly	31	50.0	31	50.0	0.522
	Sometimes/never	21	40.4	31	59.6	
	Don’t mind	5	38.5	8	61.5	
What is the most convenient time for you to exercise?	9–5 pm^a^	4	66.7	2	33.3	0.026
	Early morning	16	36.4	28	63.6	
	Early evening	12	32.4	25	67.6	
	Does not matter	24	61.5	15	38.5	
Do you feel uncomfortable exercising by yourself? (*N* = 133)	All/most/sometimes	23	43.4	30	56.6	0.488
	Never	22	51.2	21	48.8	
	Don’t mind	12	37.5	20	62.5	
Do you feel uncomfortable exercising in groups? (*N* = 127)	All/most/sometimes	16	32.7	33	67.3	0.043
	Never	21	60.0	14	14.0	
	Don’t mind	18	41.9	25	58.1	

a Fisher’s exact tests were used where cell sizes were <5. Significance levels did not differ in Fisher’s exact tests.

### Exercise advice and types

Participant exercise advice and types are presented in Table 4. The most common sources of advice on being more active were general practitioner (GP; 26%) followed by friends (19%) and Aboriginal Medical Service (AMS; 17%). Fishing (42%) was the most frequent traditional activity reported by participants, followed by swimming (33%). Almost one-third of respondents reported never participating in traditional activities (32%). Food gathering was the least frequent traditional activity (13%). Less than 20% of participants had previously had a gym membership with similar rates among males and females.

**Table 4:** Exercise advice and types by sex

Variable		Males		Females		*P*
		*N*	%	*N*	%	
Where do you get your advice on being more active? (multiple responses allowed; *N* = 133)	GP: yes	17	48.6	18	51.4	0.426
	GP: no	40	40.8	58	59.2	
	AMS: yes	8	36.4	14	63.6	0.501
	AMS: no	49	44.1	62	55.9	
	Partner: yes	9	64.3	5	37.5	0.087
	Partner: no	48	40.3	71	59.7	
	Personal trainer: yes^a^	2	40.0	3	60.0	0.875
	Personal trainer: no	55	43.0	73	57.0	
	Friend: yes	7	28.0	18	72.0	0.096
	Friend: no	50	46.3	58	53.7	
	Family: yes^a^	3	27.3	8	72.7	0.275
	Family: no	54	44.3	68	55.7	
Have you participated in any of the following traditional activities? (multiple responses allowed; *N* = 136)	Hunting: yes	16	45.7	19	54.3	0.644
	Hunting: no	41	42.3	56	57.7	
	Fishing: yes	35	46.1	41	53.9	0.572
	Fishing: no	22	40.0	33	60.0	
	Food gathering: yes	10	58.8	7	41.2	0.259
	Food gathering: no	47	40.9	68	59.1	
	Swimming: yes	14	31.8	30	68.2	0.120
	Swimming: no	43	48.9	45	51.1	
	Dancing: yes	7	33.3	14	66.7	0.590
	Dancing: no	49	45.4	59	54.6	
Have you had a gym membership previously? (*N* = 130)	Yes	14	48.3	15	51.7	0.585
	No	43	42.6	58	57.4	
Why did you join the gym?	Fitness: yes	20	45.5	24	54.5	0.792
	Fitness: no	37	43.0	49	57.0	
	Health: yes	32	41.6	45	28.4	0.526
	Health: no	25	47.2	28	52.8	
	Shape up for summer: yes	4	33.3	8	66.7	0.549
	Shape up for summer: no	53	44.9	65	55.1	
	Weight loss: yes	10	43.5	13	56.5	0.969
	Weight loss: no	47	43.9	60	56.1	

a Fisher’s exact tests were used where cell sizes were <5. Significance levels did not differ in Fisher’s exact tests.

## DISCUSSION

Understanding the facilitators and barriers to exercise is crucial for exercise programmes to be effective in improving health outcomes and to be sustainable for Indigenous people. The rationale for the focus on physical exercise is evidence-based and clear, and innovative strategies which incorporate better physiological and psychological health, building pride, cultural identity and self-esteem, are critical (Macniven *et al.*, 2019; Merone *et al.*, 2020). These findings have the potential to inform future programme development and indicate the importance of building in a level of flexibility into their design. This will allow for exercise to be done individually or in group contexts, within a gym or at home in a private space and incorporating traditional activities.

We found several differences in barriers and facilitators experienced by males and females, with more females than males reporting that daily exercise was challenging and too time-consuming.

Lack of time has been identified as a physical activity barrier in other studies with Indigenous participants (Péloquin *et al.*, 2017; Macniven and Esgin, 2020) as well as in a national Australian study (Hoare *et al.*, 2017). Similarly, another large Australian study found that ‘making time to be active’ was an enabler to physical activity (Macniven *et al.*, 2014). However, this is the first study to specifically demonstrate time as an exercise barrier among females. We consider the reasons for this to include managing multiple commitments including family and work. This underscores the value in flexibility of exercise options and of having supportive environments for exercise. We also found that proportionately more females than males reported finding vigorous exercise challenging, preferred exercising in private and at particular times of the day. This underscores the importance of facilitating feasible and culturally relevant opportunities for Indigenous women to participate in physical activity (Stronach *et al.*, 2019; Allen *et al.*, 2021). Females also expressed discomfort exercising in groups, a finding that is inconsistent with previous evidence but which highlights the benefit of a range of options to increase female exercise participation (Stronach *et al.*, 2019).

A novelty of this study is the examination of traditional physical activity preferences that indicate culturally relevant activities for future promotion and participation. Traditional activities such as fishing, swimming and hunting have played and continue to play an important role in contemporary Aboriginal culture (Gray *et al.*, 2013). Fishing, for example, was and continues to be a way of life in Noongar culture. It is still a central cultural practice, and a means to provide dietary sustenance, income and connection to culture, family and country (Gray *et al.*, 2013; Russell *et al.*, 2015). Swimming is also an important exercise in Aboriginal communities. Studies involving Aboriginal communities and swimming include prevalence or adherence studies and more recently, systematic reviews focusing on swimming pools and health outcomes (Hendrickx *et al.*, 2016). We acknowledge that some of these studies included Indigenous Peoples in different geographical and cultural contexts to the urban population in the present study, and note reports that traditional practices of hunting and burning play an important role in maintaining community cohesion, in the transfer of cultural knowledge; and in affirming both Aboriginal identities and the socio-cultural benefits of traditional hunting of dugongs and green turtles in the Torres Strait (Codding *et al.*, 2014). Increasing participation in culturally relevant physical activities is likely to have holistic wellbeing benefits beyond the physical health benefits of exercising and could be promoted for broader community and cultural benefit.

Gaining an understanding of Noongar participants’ perceptions regarding the cost associated with exercise is also pertinent as it impacts participation. Around half of the participants reported that exercise was expensive, including ‘half the time’, ‘most times’ or ‘always’. Cost has been identified as a barrier to exercise participation in another study with urban Indigenous participants (Hunt *et al.*, 2008). To increase participation, it is vital that cost is not perceived as a barrier.

Aboriginal culturally safe gyms exist in three Australian cities, Sydney (Macniven and Esgin, 2020), Melbourne and Adelaide with heavily subsidised membership rates for Indigenous people. While the absolute cost of gym membership can be an influencing factor, service quality, satisfaction and behavioural intentions are also important in gym adherence and gym membership renewals (Murray and Howat, 2002). For Indigenous participants, a culturally appropriate facility has been reported as essential (Macniven and Esgin, 2020).

It is important to develop culturally safe exercise options and facilities where Indigenous people live and to monitor whether such initiatives increase accessibility and exercise participation. To date, there have been no studies conducted on Aboriginal views of gym quality, their satisfaction with, and loyalty to a gym (Howat and Assaker, 2013) and the impact of subsidised memberships. Programmes that source affordable exercise equipment may also reduce exercise barriers among people with lower incomes (Sullivan and Lachman, 2016).

Social and structural determinants of health underlie many of the risk factors contributing to the high burden of chronic and cardiometabolic diseases among Aboriginal and Torres Strait Islander People. A national study of health system determinants, conducted principally by Aboriginal investigators with eight separate Aboriginal Medical Services and their communities identified, as critical, access to services that had the following attributes; locally defined and culturally safe, staffed by appropriately skilled culturally competent practitioners, and providers of best practice care that addresses the particular needs of a community (Davy *et al.*, 2017).

In addition, recent research into health system cultural awareness has found that the perceptions of health professionals on the key issues impacting Aboriginal and Torres Strait Islander health were significantly misaligned with the documented burden of preventable disease in these communities. The study found a need to place greater emphasis on the expansion of the Aboriginal and Torres Strait Islander health workforce and on the inclusion of pre- and in-service training of all health professionals in culturally safe health systems and practice and in Indigenous health and burden of disease subject areas (Crouch *et al.*, 2020). In the current study context, these findings together suggest that exercise physiologist/physical fitness industry workforce perceptions may impact exercise participation by Aboriginal and Torres Strait Islander Peoples.

Culturally appropriate and safe encouragement of exercise participation has the potential to convey a number of positive effects, both physically and psychologically. Physically, exercise can improve both strength and fitness, and psychologically, it has the means to deliver people from social isolation and reduce stress and depression (Macniven *et al.*, 2019; Merone *et al.*, 2020). Providing the resources or opportunity for individuals to participate in exercise may be an effective and cost-effective way to promote social and emotional wellbeing and social connection (Utting *et al.*, 2012). The preference for exercising in groups also reflects the potential to nurture a positive relationship between exercise and community cohesion. Other studies have also described improved exercise adherence and increased engagement due to social support from friends or family (Sullivan and Lachman, 2016).

In addition, recent research into health system cultural awareness has found that the perceptions of health professionals on the key issues impacting Aboriginal and Torres Strait Islander health were significantly misaligned with the documented burden of preventable disease in these communities. The study found a need to place greater emphasis on the expansion of the Aboriginal and Torres Strait Islander health workforce and on the inclusion of pre- and in-service training of all health professionals in culturally safe health systems and practice and in Indigenous health and burden of disease subject areas (Crouch *et al.*, 2020).

This current study presents new findings on facilitators and barriers to engaging in exercise among the Noongar Aboriginal community in the Perth metropolitan area, through a co-designed, culturally relevant questionnaire (Esgin *et al.*, 2019). The use of this questionnaire (Esgin *et al.*, 2019) has elicited information surrounding facilitators and barriers to exercise in the Noongar nation and the Eora nation (Macniven and Esgin, 2020) and we recommend it in either paper form or online format depending on the community’s preferences and access to technology. Advancing the establishment of a culturally appropriate gym, similar to those that exist in other Australian cities, would help to enhance the fitness and health of Noongar people living in Perth and may contribute to a reduction in the incidence and prevalence of chronic disease risk factors. Expanding the Aboriginal and Torres Strait Islander exercise and fitness workforce and training of all exercise professionals in culturally safe practice is also important.

## LIMITATIONS

A limitation of the research method is that most recruitment occurred at workplaces, meaning that the survey sample is more representative of employed Noongar adults only. Incorporating a qualitative research methodology, such as in-depth interviews or focus groups, may provide greater opportunity to elicit rich insights into the complexities of people’s relationship with physical activity and exercise. However, the strength of the quantitative questionnaire was its development with the community (Esgin *et al.*, 2019). It offers a reliable and more rapid way to elicit information on facilitators and barriers to exercise.

## CONCLUSION

Through dissemination of a questionnaire exploring the facilitators and barriers to exercise in the Noongar community, this study highlights some points for consideration when planning future exercise programmes and interventions. The Noongar community, like other communities, is diverse and there was no clear consensus for a singular approach. However, the cost of exercise was an issue and participants valued culturally welcoming venues and options for exercise at various times of day and through a variety of activities. Traditional activities and forms of exercise were very important, and exercise was viewed as a social opportunity, where being in groups and meeting with other members of the community was valued. Overall, programmes and venues need to be culturally secure, inclusive, non-judgmental and embed exercise into community approaches to promoting health and wellbeing benefits. Greater emphasis is also needed on the expansion of the Aboriginal and Torres Strait Islander exercise and fitness workforce and on the inclusion of pre- and in-service training of all exercise professionals in culturally safe practice and in Indigenous health and burden of disease subject areas.

## SUPPLEMENTARY MATERIAL

Supplementary material is available at *Health Promotion International* online.

## Supplementary Material

daac023_Supplementary_Data
