# Supplementary material for: The facilitators and barriers to exercise in the Noongar Aboriginal population in Perth, Australia
Source: Health Promot Int. 2022 Mar 28;38(3):daac023. doi: 10.1093/heapro/daac023 (PMC10308206; doi:10.1093/heapro/daac023)
Supplement: daac023_Supplementary_Data [file daac023_supplementary_data.docx]

**Study questionnaire**

1. How old are you?________or if you prefer, indicate your age by ticking one of the boxes below

18 – 25 26 – 33 34 - 40 41 – 45 45 - 50 50 +

1. Are you Male or Female
2. When was the last time you visited your doctor in relation to high blood pressure, diabetes, cholesterol or heart disease?

Within 3 months Within 6 months Within 12 months Never

1. Please tick the following box/es if you suffer from any of the following

High Blood pressure Diabetes Cholesterol Heart Disease

| Has a doctor told you, you have or are at risk of having high blood pressure? | Has a doctor told you, you have or are at risk of getting diabetes? | Has a doctor told you you have high cholesterol? | Has a doctor told you you have a heart disease? |
| --- | --- | --- | --- |

1. What is your height? _____________cm
2. What is your weight?______kg
3. What is your waist size?______cm or pants / jeans size____________?

**Barriers to exercise**

1. Where do you get your advice on being more active?

GP AMS Partner Personal trainer other ________________________

1. What injuries do you have that prevent you from exercising, if any please list

1. Is exercising vigorously more than 10 mins continuously too hard for you?

Never Sometimes Occasionally Most times Always

1. Is exercising too time consuming?

Never Sometimes Occasionally Most times Always

1. Is exercising too expensive

Never Sometimes Occasionally Most times Always

1. How difficult is the following for you?
2. Changing your diet

Very difficult Difficult Easy Very Easy

1. Finding time to exercise every day

Very difficult Difficult Easy Very Easy

1. Motivating yourself to exercise

Very difficult Difficult Easy Very Easy

1. Exercising with an injury

Very difficult Difficult Easy Very Easy

1. Putting up with the pain associated with exercise

Very difficult Difficult Easy Very Easy

1. Exercising the next day as a result of pain associated from exercising the day before

Very difficult Difficult Easy Very Easy

14. What is the most convenient time for you to exercise?

9 – 5pm Early morning Early evening Does not matter

15. Any other reasons you don’t exercise? __________________________________________________________________________________

16. What types of things might encourage or support you to exercise more regularly?

__________________________________________________________________________________

**Exercise habits and environment**

17 . Do you feel uncomfortable exercising by yourself?

- All times Most Sometimes Never Don’t mind

If so why? _________________________________________________________________________

18. Do you prefer to exercise

- On your own All times Most Sometimes Never Don’t mind
- in small groups (2-6) All times Most Sometimes Never Don’t mind
- in medium groups (7 +) All times Most Sometimes Never Don’t mind

19. Do you feel uncomfortable exercising in groups?

All times Most Sometimes Never Don’t mind

If so why? _________________________________________________________________________

20 a. Do you prefer to exercise in

public or private or both

Indoors or outdoors or both

21. Do you enjoy playing games involving sports equipment (i.e Football, Netball, Rackets, Golf )?

All times Most times Sometimes Never Don’t mind

22. Do you do any of the following traditional physical activities?

Hunting Fishing Food gathering Swimming Dancing

23. Would you like to try any of these activities to improve your fitness and activity levels?

Please rate each one of these activities from 1 – 5. 5 being an activity you are interested in and 1an activities you are not interested in.

Walking / Walking group ______ Score 1 - 5

Dancing (Salsa) ______ Score 1 - 5

Self defence training ______ Score 1 - 5

Aerobics (Zumba or Pump classes) ______ Score 1 - 5

Gym (lifting weights and running on treadmills) ______ Score 1 - 5

Gym (circuit training) ______ Score 1 - 5

Boot camp (training at parks and beaches) _______ Score 1 - 5

Team ball sports (Footy, Netball, Volleyball) _______ Score 1 - 5

Traditional Indigenous activities / games _______ Score 1 - 5

24. Do you own any of these exercise machines?

Treadmill Stability Ball Home Gym Dumbbells

Exercise bike Skipping Rope Stepper Fitness DVD’s

Other___________________________________________________________________________

25. Have you had a gym membership before? Yes No

If not why not? _______________________________________________________________

If yes, how often do you go to the gym? ________________

Why did you join the gym? Fitness Shape up for summer

Health Weight loss

26. Why did you quit the gym? Culturally inappropriate Cost too much

Not enough time Too tired

Gym equipment to confusing Gym outfits expensive

Other_________________________________________________________________

27. What was your favourite activity in the gym?

Using weights by yourself Pump class Aerobics Aqua Aerobics

Other_________________________________________________________________

28. If you were able to attend a gym that was flexible enough to meet your and the community’s needs with other Indigenous participants and have access to a personal trainer all for free, would you be willing to train in a gym?

Yes No Maybe

Other comments– Please also indicate what suburb is most suitable for you to attend the exercise setting __________ _____________________________________________________________

_______________________________________________________________________________

29. Goals

1. Please list in order of priority 3 physical activity based goals you would like to achieve over the next 3-6 months? Eg being able to double the amount of exercise or running 2.4 km in 12 min.
2. ________________________________________________________________________

b)_________________________________________________________________________

c)_________________________________________________________________________

30. How committed are you to achieving fitness goals? Please circle a number

Very Committed Semi Committed Not very Committed

1 2 3 4 5

Outline any obstacles, potential actions, behaviours or activities that could limit your progress towards accomplishing your goals (i.e. not training consistently, upcoming vacation, busy season at work, not following the program, allowing other responsibilities tobecome a priority over exercise etc.).

___________________________________________________________________________

___________________________________________________________________________
